# Development of Antioxidant and Stable Conjugated Linoleic Acid Pickering Emulsion with Protein Nanofibers by Microwave-Assisted Self-Assembly

**DOI:** 10.3390/foods10081892

**Published:** 2021-08-15

**Authors:** Qiyang Jiao, Ziyuan Liu, Baoyun Li, Bo Tian, Ning Zhang, Chunhong Liu, Zhibiao Feng, Bin Jiang

**Affiliations:** 1 Department of Chemistry, College of Arts and Sciences, Northeast Agricultural University, Harbin 150030, China; jiaoqiyang0729@126.com (Q.J.); liuziyuan11AB@163.com (Z.L.); libaoyun129@163.com (B.L.); liuchunhong@neau.edu.cn (C.L.); 2 College of Food Science, Northeast Agricultural University, Harbin 150030, China; tianbot@163.com; 3 Key Laboratory of Mineral Resources and Ecological Environment Monitoring, Baoding 071051, China; nzhangstar@163.com

**Keywords:** whey protein isolates nanofibrils, microwave heating, Pickering emulsion, conjugated linoleic acid

## Abstract

Whey protein isolate nanofibrils (WPNFs) can be used as a novel stabilizer in the Pickering emulsion system to improve the water solubility, stability and bioavailability of lipophilic bioactive ingredients. In this study, conjugated linoleic acid (CLA) and WPNFs were used to prepare a stable Pickering emulsion. We used a transmission electron microscope, low-temperature scanning electron micrographs and other methods to evaluate the micromorphology, surface hydrophobicity and structural units of the obtained WPNFs. Compared with whey protein isolate/CLA Pickering emulsion, the WPNFs/CLA Pickering emulsion has greater ability to remove 2,2-Diphenyl-1-picrylhydrazyl and 2,2′-amino-di(2-ethyl-benzothiazoline sulphonic acid-6) ammonium salt free radicals. Furthermore, the WPNFs/CLA Pickering emulsion has a more stable effect in terms of droplet size and zeta potential over a wider range of ionic strength and temperature conditions. These findings indicate that Pickering emulsion stabilized by WPNFs is more suitable as a carrier of CLA, as it increases the solubility of CLA and has better active applications in biology and food.

## 1. Introduction

Protein nanofibrils (PNFs) are unique self-assembled aggregates known for their well-ordered fibrous morphology, high aspect ratio, high flexibility, biodegradation and biocompatibility [1]. PNFs with improved functional and biological properties can be used as emulsifiers and thickeners and for food packaging and coatings in the food industry [2,3]. PNFs are intrinsically insoluble particles with intermediate wettability [4,5,6] and have the ability to irreversibly adsorb at the interface between the oil and water phases to form a stable physical protective layer [7]. Furthermore, PNFs have no direct toxicity to human cells in vitro and can be utilized as edible biomaterials without safety concerns. Thus, PNFs were introduced to stabilize Pickering emulsions as a new food delivery system. Fibrillation is accompanied by the improvement and creation of new structures, enabling proteins to obtain improved physicochemical and functional properties, such as antioxidant activity, surface hydrophobicity, emulsifying activity and gelling properties, which cannot be obtained by other methods [3,8]. It is important to note that different original protein and preparation conditions will produce distinctive PNFs with different morphologies, functional properties and applications. Thus, PNFs prepared with abundant original resources, simple preparation methods, a specific structure and functional properties can help to realize large-scale commercial applications [5]. 

In addition to traditional molecular emulsifiers, solid particles can be used as emulsion stabilizers; particle-stabilized emulsions are known as “Pickering emulsions” [9]. Pickering emulsions have important applications in many areas due to their low cost and because they are non-toxic and exhibit environmentally friendly properties [10]. Conjugated linoleic acid (CLA) was chosen to be encapsulated in Pickering emulsion because it has many disadvantages of lipophilic bioactive ingredients (LBI), such as poor water solubility, poor chemical stability and high oxidation sensitivity, and is easy to decompose and oxidize during processing, storage and digestion. These deficiencies make it difficult to use CLA as a food ingredient [11]. Nevertheless, CLA has numerous potent physiological activities, such as anti-cancer, anti-inflammatory, anti-atherosclerosis, anti-diabetes and anti-hypertension effects [12]. Encapsulating CLA in a Pickering emulsion system is therefore of potential practical significance.

The properties of PNFs meet the basic prerequisites of being a Pickering stabilizer, and PNFs-stabilized Pickering emulsions are extremely stable under a wide range of pH, ionic strength and temperature conditions [7]. PNFs have advantages in drug control due to their sustained release and targeted nutrient delivery. Whey protein isolate (WPI), a byproduct of the cheese industry and casein, is highly nutritious and has many biological functions [13,14]. Consequently, whey protein isolate nanofibrils (WPNFs) have attracted attention because they can be used as a stabilizer in Pickering emulsions to encapsulate various hydrophobic bioactive compounds, such as orange peel oil, micronutrients and curcumin, which can improve their solubility, bioactivity and chemical stability [15,16,17]. Compared with conventional protein–polysaccharide particle systems, PNFs are popular because they are easy to prepare and have better biostability under a wider range of environmental conditions [18].

Most preparation methods for PNFs are focused on a conventional heating method—that is, heating acidified protein solutions (commonly at pH 2.0) at over 80 °C for more than 10 h [19]. Microwave-assisted self-assembly (MASA) is the process of using the energy characteristics of microwaves to heat objects. Microwave irradiation can directly induce torsional vibrations in the protein backbone, and the secondary and tertiary structures of proteins are altered by denaturation and refolding [20,21]. On the other hand, MASA can selectively break peptide bonds and form small peptides of terminal aspartic acid that are the building blocks of PNFs [22,23,24]. Therefore, it may be possible to use MASA to accelerate the formation of PNFs rapidly. Hettiarachchi et al. reported that *β*-lg nanofibers could be prepared by MASA, which was different from the conventional heating [22]. Lee further investigated the formation of oligomeric amyloid aggregates under microwave irradiation conditions and found that amyloid oligomers were formed within 7 min [20]. In our previous study, WPI formed WPNFs in 4 h using MASA [4]. Compared with conventional heating methods, MASA can be used to obtain the same or better quality fibers in a shorter period of time.

Accordingly, the aim of this study was to evaluate the ability of WPNFs prepared by the MASA method to form Pickering emulsions as a carrier for CLA in order to develop an effective method to increase the solubility of CLA. To achieve this, we evaluated the micromorphology, surface hydrophobicity and building blocks of the prepared WPNFs. The microstructure, emulsion characteristics, emulsion stability against ionic strengths and heating and antioxidant activity of the WPNFs/CLA Pickering emulsion were investigated. 

## 2. Materials and Methods

### 2.1. Materials

WPI (protein content > 91.5%) was acquired from Hilmar Industries (Hilmar, CA, USA). Thioflavine T (ThT), 8-anilino-1-naphthalenesulfonic acid (ANS), Congo red, 1,1-diphenyl-2-picrylhydrazyl (DPPH) and 2,2′-azino-bis-3-ethylbenzthiazoline-6-sulfonic acid (ABTS) were purchased from Sigma (St. Louis, MO, USA). All other chemicals and reagents were higher than analytical grade.

### 2.2. Preparation of WPNFs by MASA

WPNFs were prepared by reference to our previous experimental method [4]. After the pH was adjusted to 2.0 with 3 mol/L HCl solution, the 3% WPI solution (*w*/*v*) was stirred at room temperature (20–25 °C) and allowed to hydrate completely. The solution was centrifuged at 9000× *g* for 15 min at 4 °C (Z236HK Hermle, Wehingen, Germany), and the membrane was then filtered under vacuum using a mixed fiber membrane with a pore size of 0.45 μm. The filtrate was subjected to microwave digestion apparatus (XH-800C, Beijing, China) for MASA at 80 °C and 800 W. The control group was prepared as a 3% WPI (*w*/*v*) solution without MASA.

### 2.3. Transmission Electron Microscopy (TEM)

The solution of WPNFs was diluted 10-fold with HCl solution (pH 2.0) and placed in a filter centrifuge tube (100 kDa) and ultrafiltered at 3000× *g* for 10 min, and this procedure was repeated three times [6]. The filtrates were then recovered and combined and stained negatively with 2% uranyl acetate, and the micrograph of WPNFs was performed using an H-7650 transmission electron microscope with Olympus imaging system (Hitachi, Tokyo, Japan).

### 2.4. Congo Red Spectroscopic Assay of WPNFs Formation

Congo red spectroscopy was used to monitor the preparation process of WPNFs, with some modifications to the method in Jiang et al. [25]. The WPNFs solution (500 μL) and Congo red solution (pH 7.0, 5 mL, 100 μM Congo Red, 10 mM phosphate, 150 mM NaCl) were mixed thoroughly and left to stand at 20–25 °C for 30 min. After that, the absorption spectra were monitored with an UV-2100 spectrophotometer (Shimadzu, Tokyo, Japan) at 400–600 nm three times.

### 2.5. Determination of Surface Hydrophobicity

ANS fluorescence intensity can be used as an indicator for the level of surface hydrophobicity of protein [26]. A total of 4 mL of WPNFs solution was diluted with HCl solution (pH 2.0) to 0.01% (*v*/*v*). The diluent was mixed with ANS solution (20 μL, 8.0 mmol/L) and shaken for 3 min. The fluorescence spectra were determined using an LS-55 fluorescence spectrometer (Perkin Elmer, Boston, MA, USA) at a wavelength range of 400–600 nm (excitation wavelength of 370 nm, slit width of 10 nm, scanning speed of 500 nm/min). Surface hydrophobicity was calculated as the initial slope in a linear regression curve of the sample protein concentration and the corresponding fluorescence intensity.

### 2.6. Liquid Chromatography-Tandem Mass Spectrometry (LC-MS/MS)

WPNFs were depolymerized at pH 10.0 and analyzed by LC-MS/MS. Solution A was an aqueous solution of 0.1% formic acid and solution B was a mixture of 0.1% formic acid, acetonitrile and water (where acetonitrile is 95%). The liquid chromatographic column (75 µm × 150 mm, RP-C18, Column Technology Inc., Folsom, CA, USA) was balanced with 95% mobile phase A. The peptides mixture was injected into a Zorbax 300SB-C18 peptide trap (Agilent Technologies, Wilmington, DE, USA) and then separated on the balanced chromatographic column. The peak was analyzed by a Q-Exactive mass spectrometer (Thermo Fisher, Waltham, MA, USA). The MS method consisted of a cycle combining one full MS scan with two MS/MS events (25% collision energy). The dynamic exclusion duration was set to 30 s [14].

### 2.7. Pickering Emulsion Fabrication

The WPNFs/CLA Pickering emulsion (WFPE) was prepared by mixing 15 mL of CLA with 85 mL of WPNFs solution using a high-shear mixer (ESB-500, ELE Company, Shanghai, China) at 10,000 rpm for 2 min at 25 °C. The emulsion was then sonically dispersed at 400 W for 15 min to achieve homogenization. The WPI/CLA Pickering emulsion (WPE) was prepared with 3% WPI solution as a control.

### 2.8. Confocal Laser Scanning Microscope (CLSM)

Confocal laser scanning microscopy (Leica Microsystems Inc., Heidelberg, Germany) was used to evaluate the microstructure of the emulsion derived from the previous experiment, with some modifications [27]. A total of 40 μL of the fluorescent dye Nile red (0.1%, *w*/*v*) and ThT (0.01%, *w*/*v*) were mixed thoroughly to make a fluorescent stain, and 1 mL of the sample was stained. We placed the stained emulsion between the concave slide and the coverslip, and the detection was then carried out at the excitation wavelengths corresponding to Nile red (488 nm) and ThT (408 nm). 

### 2.9. Super Resolution Microscopes (SRM)

Super resolution experiments were based on the method in Chen et al., with some modifications [28]. A 0.5% (*w*/*v*) Nile red solution was prepared with isopropanol as solvent. Then, 1 g emulsion sample was diluted with 50 mL of 0.01 mol/L HCl solution to obtain the first diluent, and 1 mL of the first dilution was mixed with 4 mL of 0.01 mol/L HCl solution to obtain the second diluent.

A total of 1 μL of the 1 mg/mL rhodamine B isothiocyanate staining solution was added to the second dilution, and this was then mixed and stained for 60 min protected from light. The prepared Nile red staining solution was then added and mixed and stained for 20 min protected from light. Following this, 20–30 µL stained emulsion was dropped onto a clean coverslip, the slide was placed on the coverslip and the excess sample was gently blotted off with filter paper. Afterwards, the samples were sealed. Microstructure analysis was performed using a GE DeltaVision OMX SR super resolution microscope (OMX SR, Leica, Wetzlar, Germany) with a light source of argon krypton laser (ArKr 488 nm) and rhodamine B isothiocyanate fluorescence excited at 568 nm. The oil phase was stained green and WPNFs were stained red in the images.

### 2.10. Cryogenic Scanning Electron Micrograph (Cryo-SEM)

The micromorphology of the emulsion was investigated with Cryo-SEM. The sample was filled into the sample tank and rapidly immersed into liquid nitrogen to crystallize and was then transferred to the freezing chamber connected to the electron microscope. The sample was kept at –90 °C for 10 min and then coated with gold. The micrograph was obtained by SEM (H-7650, Hitachi, Tokyo, Japan) under high pressure vacuum and 5 kV accelerating voltage.

### 2.11. Droplet Size Distribution and Zeta Potential

The droplet size distribution and zeta potential of the emulsions were measured using a Malvern Nano-S90 laser particle size analyzer (Malvern Instruments Inc., Malvern, UK) derived from the experimental method of Feng et al. [29]. Prior to measurement, a dilution process was required, during which the emulsion was diluted 1000 times with HCl (pH 2.0) and set aside.

### 2.12. Emulsifying Activity and Emulsion Stability

In terms of emulsification properties, the emulsification activity index (EAI) and emulsion stability index (ESI) were used as important reference indicators, determined using the method from [30] with slight modifications. Briefly, 10 mL of 0.1% sodium dodecyl sulfate (SDS) solution was mixed with 5 μL of emulsion. We used a 0.1% SDS solution as a blank, and the absorbance of the sample was monitored at an absorption wavelength of 500 nm. The EAI value was calculated according to the following equation:

(1) $$\mathrm{EAI}\left(m^{2}/g\right)=\frac{2\times 2.303{\times A}_{0}\times N}{c\times \Phi \times 10,000}$$

where *A*_0_ represents the absorbance at 0 min, *N* is the dilution factor, *c* (g/mL) is the concentration of WPNFs and *Φ* is the oil volume fraction (15%).

ESI was calculated as follows:

(2) $$\mathrm{ESI}\left(\min\right)=\frac{A_{0}}{A_{0}-A_{1}}\times t$$

where *A*_0_ and *A*_1_ are the absorbance at 0 min and 30 min, respectively, and *t* is the time interval.

### 2.13. Environmental Stress Stability

The stability of the emulsion was determined when exposed to a range of ionic strength and temperature conditions on the basis of Jiang et al. [31]. We diluted the emulsion samples 100-fold using 100, 200, 300, 400 and 500 mM NaCl solution, and they were centrifuged at 4000 rpm for 15 min. The absorbance of the bottom solution was monitored at 500 nm with a spectrophotometer. The stability of emulsion under different ionic strengths was evaluated by the centrifugation stability constant Ke. The formula to calculate this is as follows:

(3) $$\mathrm{Ke}=\frac{\left|A_{0}-A_{1}\right|}{A_{0}}\times 100$$

where *A*_0_ and *A*_1_ are the absorbance of the emulsion before and after centrifugation, respectively.

Temperature stability was determined by keeping samples at different temperatures (30, 40, 50, 60, 70 and 80 °C) for 15 min. After heating, they were cooled to room temperature (25 °C). The centrifugation stability constant Ke of the emulsion was monitored.

### 2.14. DPPH Radical Scavenging Activity Assay

The DPPH radical scavenging activity of emulsions was determined according to the method of Jiang et al. [32]. Specifically, 0.1 mL of emulsion and 4.0 mL of DPPH solution (0.1 mM) that had been prepared in 95% ethanol were mixed thoroughly, and the solution was kept in the dark at 25 °C for 30 min. Afterwards, the absorbance was monitored at 519 nm using a UV-2500 spectrophotometer (Shimadzu, Tokyo, Japan). The blank set was an ethanol (95%) solution without DPPH, and ultrapure water replaced the sample as a negative control. The DPPH radical scavenging activity (DPPHscav) was calculated using the following equation:

(4) $${\mathrm{DPPH}}_{\mathrm{scav}}\%=(1-\frac{A_{\mathrm{sample}}-A_{\mathrm{control}}}{A_{\mathrm{blank}}})\times 100$$

### 2.15. ABTS^+^ Radical Scavenging Activity Assay

The ABTS^+^ assay was based on the experimental method of Feng et al., with modifications [6]. The ABTS^+^ solution was diluted with phosphate buffer (pH 7.4) to adjust its absorbance to 0.7 ± 0.02 at 734 nm. A total of 30 µL emulsion was mixed with 3 mL ABTS^+^ radical cation (ABTS^+•^) solution, and the absorbance at 734 nm was measured at 0.5, 1, 1.5, 2, 2.5 and 3 min for each of the sample reactions (Shimadzu spectrophotometer UV-2500, Tokyo, Japan). The blank control consisted of 30 μL of distilled water mixed with 3 mL of ABTS^+•^ solution. The clearance rate for ABTS^+^ was calculated using the following equation:

(5) $$\mathrm{scavenging}\mathrm{activity}\%=\frac{A_{0}-\bar{A_{i}}}{A_{0}}\times 100$$

where *A*_0_ and $\bar{A_{i}}$ are the absorbance of the blank and the sample, respectively.

### 2.16. Statistical Analysis

Mean values and the standard deviations (means ± SD) of the data were analyzed using SPSS 20.0 software (Chicago, IL, USA). Statistically significant differences between sample means (*p* < 0.05) were established using Duncan’s multiple range algorithms.

## 3. Results and Discussion

### 3.1. TEM Micrograph of WPNFs Prepared by MASA

**Figure 1:**
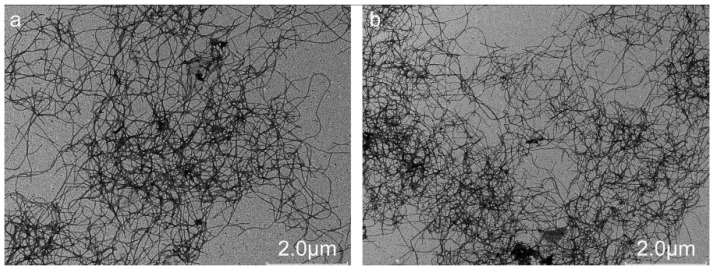
Transmission electron microscopy image of whey protein isolate nanofibrils prepared by (**a**) the conventional method and (**b**) microwave heating.

MASA can greatly shorten the preparation time of WPNFs compared with the 10 h conventional heating method [6,33]. After 4 h of heating, WPNFs prepared by MASA showed the same mature state as those prepared by the conventional heating method for 10 h. A large number of long fibers were entangled with each other, with many branches, clear shapes and clear network structures (Figure 1a,b). This result is consistent with the findings of Zhang et al. [4]. In addition, there are many high performance examples of food protein amyloid fibrils in the structuring and functionalization of food products including transparent gels, which have high elasticity and low critical gelation concentration, emulsions stabilized against heat and salt and various foods with amyloid-enhanced bioactivity [34]. Mature fibers are extremely diverse in form and include twisted ribbons, helical ribbons, nanotubes and crystal polymorphs [35]. Order transitions have been observed among these fibrils in a number of different systems and given a precise energy landscape description [36]. The twisted ribbon polymorph is the most commonly observed form of amyloid fiber, which can be transformed into a helical ribbon polymorph by a gradual increase in width (i.e., a lateral increase in the original filament). Theoretically, the helical ribbon continues to transform into nanotubes and crystalline polymorphs by reducing the free energy, but in practice, the fiber only needs to reach the twisted ribbon polymorph to become a more stable form.

### 3.2. Congo Red Spectral Analysis

**Figure 2:**
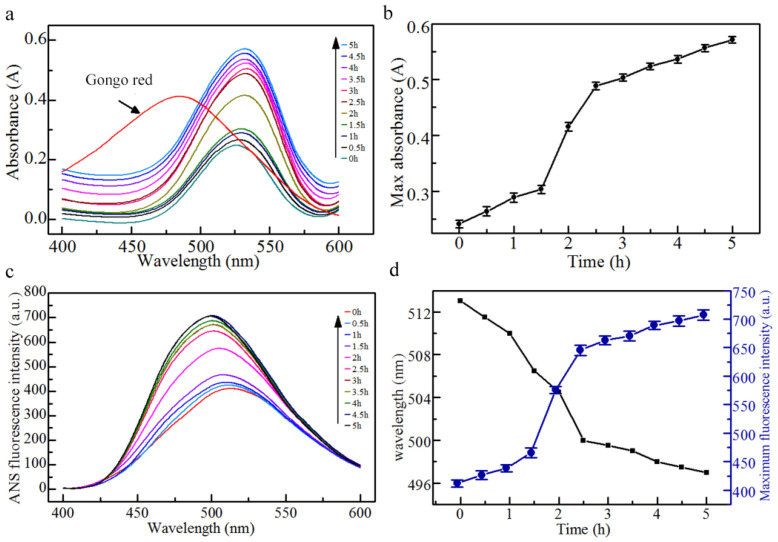
Spectra of WPNFs combined with Congo red and 8-anilino-1-naphthalenesulfonic acid (ANS). (**a**) Absorbance spectra of Congo red combined with WPNFs; (**b**) absorbance intensity of Congo red combined with WPNFs at 540 nm; (**c**) ANS intrinsic fluorescence spectra of WPNFs; and (**d**) ANS fluorescence peak and intensity at the maximum of the emission curve.

The specific binding of Congo red to amyloid fibrils results in a shift of the red absorption peak and an increase in absorption intensity [37]. The change in absorption intensity can be used to characterize the formation process of WPNFs. After Congo red was combined with WPNFs, the red absorbance peak shifted from 490 nm to 540 nm (Figure 2a). The absorbance increased with increasing microwave heating time, indicating that time is one of the factors affecting the formation of WPNFs (Figure 2b). The formation process of WPNFs could be divided into three time-dependent stages: the lag stage, growth stage and post-ripening stage. The absorbance increased slightly in the lag phase (0–1.5 h), when the WPNFs building blocks gradually accumulated. Then, the absorbance increased rapidly (1.5–2.5 h), at which point sufficient WPNFs building blocks rapidly accumulated to form WPNFs. After 2.5 h, the absorbance increased slowly. During this process, the fibrillation process entered the post-ripening stage, and the fibrils were reversibly and tightly bound together. 

### 3.3. Surface Hydrophobicity

Since the surface hydrophobicity of WPNFs is closely related to their function and application, it is valuable for understanding its surface properties. As a commonly used hydrophobic probe, ANS was used to evaluate changes in hydrophobicity during the formation of WPNFs [26]. As the ANS bound to the protein, the emission fluorescence intensity was enhanced, and the fluorescence wavelength was blue-shifted (Figure 2c,d). The hydrophobicity of the surface increased rapidly after a slow start within 0–2.5 h, and increased slowly after 2.5 h, which coincided with the three stages of Congo red spectral analysis. It is therefore reasonable to assume that the reason for the higher surface hydrophobicity of WPNFs is the exposure of hydrophobic patches that accompany its formation. This assumption is consistent with the results of Mohammadian et al. [8]. Based on the results of the surface hydrophobicity measurements, the reason for the higher binding capacity may be the easier formation of inter- and intra-molecular links between the WPNFs and the oil [38].

### 3.4. Building Blocks of WPNFs Prepared by Microwave Heating

**Figure 3:**
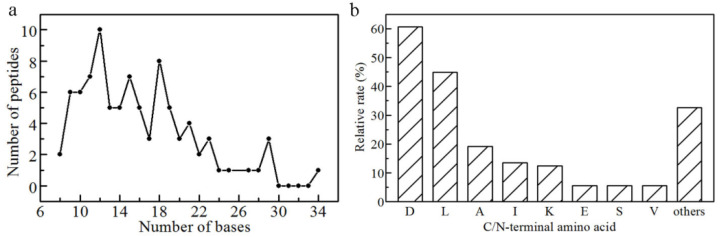
Features of identified building blocks of WPNFs. (**a**) Length distribution of identified building blocks of WPNFs; (**b**) the relative rate of different amino acids as C/N-terminal amino acid of these peptides: aspartates (D), leucine (L), alanine (A), isoleucine (I), lysine (K), glutamic acid (E), serine (S), valine (V).

In order to characterize the building blocks of WPNFs, the amino acid sequence of WPNFs was determined by LC-MS/MS. The original mass spectrometry data were checked by Proteome Discoverer in UniProt protein database (species setting cattle) (Table S1). The identified building blocks of WPNFs consisted of 89 peptides, of which 4 were from *α*-Lipoic acid and 85 were from *β*-lactoglobulin. The lengths of the peptide chains of the identified WPNFs are shown in Figure 3a. Numerous identified short peptides confirmed that WPNFs were composed of hydrolysates rather than original proteins. Peptides 9–19 bases in length accounted for 75% of those identified, indicating that peptides with a suitable length were flexible in solution to facilitate aggregation. In addition to the sequences identified for the first time, some were consistent with those identified by Akkermans [39].

Figure 3b illustrates the relative rate of the C/N-terminal amino acid in these peptides. In particular, 60.7% were terminal aspartate (D), and 44.9 % were terminal leucine (L), indicating that most of the possible building blocks might be produced by *β*-lg cleaved at the X-D or D-X bond, which is consistent with the results of Akkermans [39]. This provided further evidence that microwave heating can cleave the X–D or D–X bond of proteins in a short time, thereby effectively completing the accumulation of building blocks of WPNFs and shortening the preparation time.

**Figure 4:**
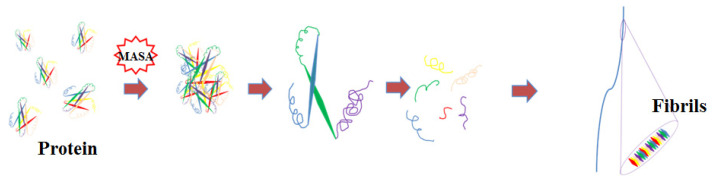
The theoretical path of fiber formation under microwave conditions.

Under microwave heating conditions, protein molecules aggregate at low temperatures and then hydrolyze into polypeptides, forming mature fibers after 4–5 h. The fiber formation process is shown in Figure 4. Compared with the traditional method, microwave heating saved a lot of time. We speculate that the existence of a high-intensity electric field in microwave heating might affect the *β*-sheet in the secondary structure of the protein, leading to the aggregation of a large amount of protein. In a certain range of conditions, the protein concentration increases rapidly, thus improving the fiber formation rate.

### 3.5. Emulsion Micromorphology by CLSM

**Figure 5:**
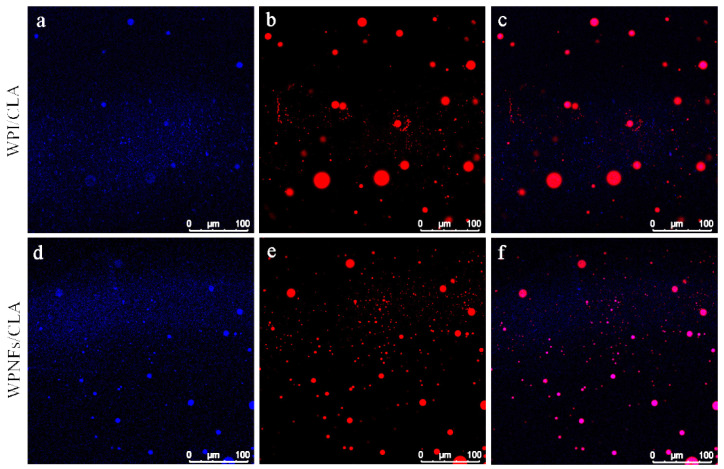
Confocal laser scanning microscope images of different samples (scale bar: 100 μm). (**a**) Whey protein isolate (WPI) and (**d**) WPNFs in emulsions, (**b**,**e**) conjugated linoleic acid (CLA) in emulsions, (**c**,**f**) overlap of fluorescence field of emulsions. WPI and WPNFs stained with Thioflavine T, blue at 408 nm and CLA stained with Nile red, red at 488 nm.

CLSM is a powerful characterization method for determining the microstructure of emulsion without destruction. The oil was stained with Nile red and are represented as red regions. ThT is a special probe that can bind to amyloid fibrils and generate a specific fluorescent signal. Therefore, WPNFs were stained with ThT and are represented as blue regions in Figure 5. Although the droplets of WPE and WFPE were well separated and no apparent coalescence occurred, the significant difference in microstructure between WPE and WFPE could be observed easily. The droplet size of WFPE was smaller than that of WPE. As shown in Figure 5, the liquid droplet appeared purple, indicating that blue WPNFs were located around the red oil droplets and that they could be used as oil-in-water Pickering emulsifiers. This was due to the increased flexibility and hydrophobicity of WPNFs. WPNFs with good flexibility had the ability to bend over the surface of the emulsion droplet to form a barrier. Furthermore, the excellent hydrophobicity of WPNFs could avoid aggregation and maintained the emulsion in a stable state.

### 3.6. Emulsion Micromorphology by SRM

**Figure 6:**
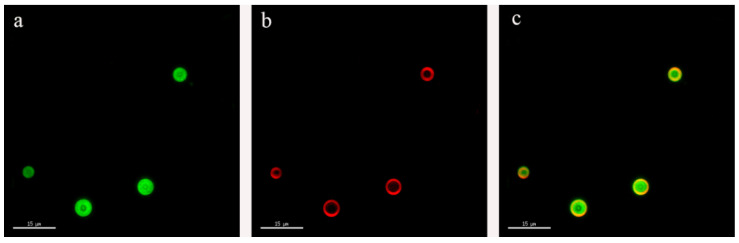
SRM image of the sample (scale bar: 15 μm). (**a**) Oil was stained with Nile red, and (**b**) WPNFs were stained with rhodamine isothiocyanate B. (**c**) Microscope images were obtained to show the overlapping fluorescent fields from (**a**,**b**). Nile red and rhodamine B isothiocyanate were simultaneously fluorescently excited at 488 nm and 568 nm, shown as green and red in the image, respectively.

Figure 6 shows the super resolution image of the emulsion sample. The oil droplets were stained with Nile red, which appears green in the image. WPNFs were stained with rhodamine isothiocyanate B, which appears red in the image. The more intense the red color, the higher the amount of WPNFs. As shown in Figure 6b, the samples showed a distinct red color and almost completely wrapped the green area shown in Figure 6a, indicating that WPNFs combined well with oil droplets. These results indicate that WPNFs can effectively prevent the collision and aggregation of oil droplets and that WPNFs participate in the formation of Pickering emulsion.

### 3.7. Emulsion Micromorphology by Cryo-SEM

**Figure 7:**
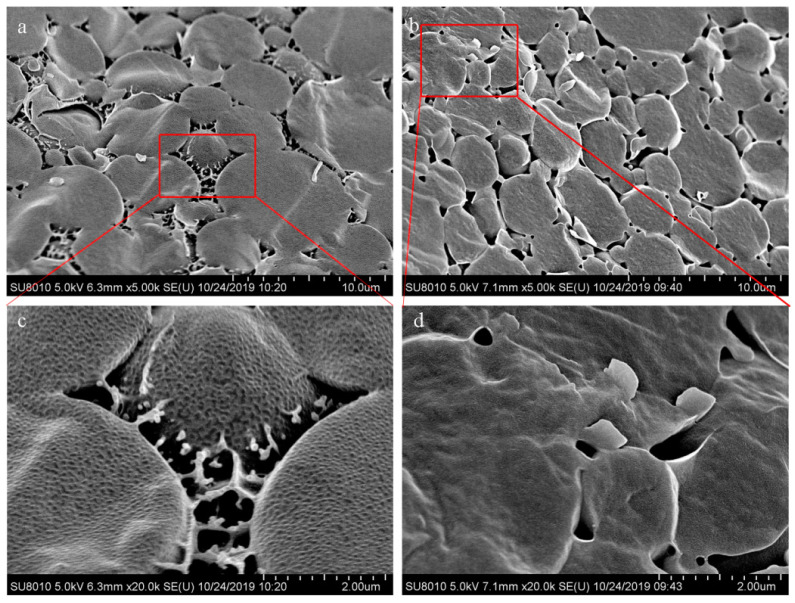
Cryogenic scanning electron micrograph images of emulsions: (**a**) WPI/CLA Pickering emulsion (WPE) (scale bar: 10 μm) and (**b**) WPNFs/CLA Pickering emulsion (WFPE) (scale bar: 10 μm) and amplified CLSM images of (**c**) WPE (scale bar: 2.00 μm) and (**d**) WFPE (scale bar: 2.00 μm).

The Cryo-SEM images of the emulsions are shown in Figure 7. The droplet structures of both WPE and WFPE were relatively intact and did not collapse. In the amplified surface morphology of WPE (Figure 7c), the surface of the WPE droplet was coated with a dense layer formed by WPI protein nanoparticles, in accordance with Persson’s results [40]. Interestingly, the structure of WFPE and WPE is consistent with the micellar structure of the particles prepared by Wiacek, i.e., the tightly packaged circular regions (cores) were surrounded by the crown consisting of radially located scaffolding layers (shells) [41]. Nevertheless, some of the boundaries between WPE droplets tended to join and disappear, which might be the main cause of poor stability. WPNFs formed a smoother layer (Figure 7d), which was significantly different from the tight arrangement of protein nanoparticles on the surface of WPE droplets. The obvious and clear boundaries between the droplets made WFPE more stable than WPE [42]. Compared with the original protein, PNFs with a proper aspect ratio and flexibility could form stable and viscoelastic interfacial layers, which formed the physical barrier to prevent coalescence during emulsification [34,43,44]. Meanwhile, due to the increased surface hydrophobicity, WPNS can irreversibly adsorb to the interface and tightly wrap the oil phase, so that the droplet looks more like a regular ball and its size is smaller [38].

### 3.8. Droplet Size and Zeta Potential

**Figure 8:**
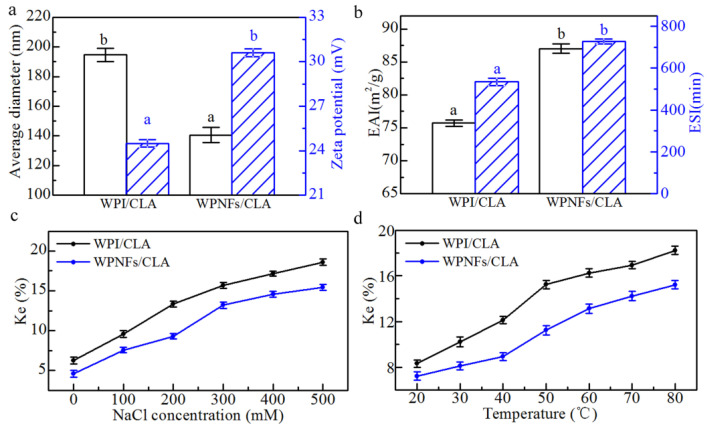
Dynamic light scattering, emulsifying properties and emulsion stability of the emulsions. (**a**) Average particle diameter and zeta potential. (**b**) Emulsification activity (EAI) and emulsion stability (ESI) of the emulsions. (**c**) Effect of NaCl concentration on Ke of Pickering emulsions. (**d**) Effect of temperature on Ke of Pickering emulsions.

Zeta potential is an important reference in both assessing emulsion stability and determining the choice of different wall materials to prepare micro- and nano-emulsions [45]. Generally, emulsion stability has been associated with zeta potential values of ± 30 mV [45]. The zeta potential values of WFPE droplets reached 30.60 ± 0.27 mV, higher than the 24.51 ± 0.26 mV of WPE droplets (Figure 8a). A higher zeta potential represents a stronger electrostatic repulsion between emulsion droplets, and the separation distance between particles is consequently greater, resulting in a more stable emulsion. 

Consistent with the results of CLSM and SRM, in Cryo-SEM, the average diameter of WFPE droplets was smaller than that of WPE (Figure 8a). WPNFs have a better binding capacity for oil due to the enhanced hydrophobicity of the surface and the exposure of charged groups caused by fibrillation, so that a spatial site-resistance stabilization effect occurs when droplets are in close proximity to each other [4,46]. Due to the more positively charged patches exposed to fibril surfaces, WPNFs had higher zeta potential than WPI [29], meaning WPNFs provide more charges on the surface of oil droplets to stabilize the emulsion. The increased surface charge density and lower average diameter of WFPE improves its stability, giving it wider potential applications in the food sector [47].

### 3.9. Emulsifying Properties

The ability of proteins to stabilize emulsions by rapidly adsorbing onto the surface of newly formed droplets is often used to define emulsification properties. The droplet size of emulsion has a great influence on the stability of an emulsion. Emulsions in which the droplet size can be precisely controlled show better stability [48]. Proteins reduce the interfacial tension at the oil–water interface through a process of physical adsorption on the surface of oil droplets. CLA is insoluble in water and soluble in organic solvents such as ethanol. However, in WPE and WFPE, due to the action of WPI and WPNFs, more CLA is dissolved in water, which increases its solubility. Figure 8b indicates that WFPE exhibited better emulsifying activity and emulsion stability than WPE. The structure of a protein is closely related to its ability to adsorb to oil and water surfaces, as well as being related to its surface hydrophobicity. As linear molecules with a higher aspect ratio, greater flexibility and surface hydrophobicity, WPNFs could effectively diffuse to newly formed interfaces and improved the emulsifying activity [29]. Additionally, higher zeta potential and the smaller droplet size contribute to an appropriate EAI and ESI of WFPE, as indicated previously.

### 3.10. Influence of Ionic Strength and Temperature on Pickering Emulsion Stability

According to previous reports, the physical stability of many protein-stabilized Pickering emulsions might be reduced at higher ionic strength, resulting in a tendency toward aggregation and particle precipitation [49,50]. The reason for this phenomenon is that the electrostatic repulsion between the oil droplets is reduced by the addition of salt to the system [51,52]. We investigated the effect of ionic strength on the stabilization of emulsions. As shown in Figure 8c, the Ke of WPE and WFPE was evaluated at different concentrations of NaCl (0–500 mM). The Ke of both emulsions was destabilized with increasing salt concentration, which was mainly because the electrostatic repulsion between the protein-coated droplets was weakened by the increase in ionic strength. In particular, under the same ionic strength, WFPE was more stable than WPE. This could be explained by the higher zeta potential and smaller average particle size of WFPE, which improved the centrifugal stability. In addition, Kowalczyk’s research shows that different ionic systems may influence emulsion stability because the stability is dependent on the kind of environment (electrolyte concentration and ions valence) [53].

The thermal stability of WPE and WFPE was analyzed using the Ke values at different temperatures (20–80 °C) (Figure 8d). The droplet shape of the Pickering emulsion can change considerably as the temperature increases and the water in the droplet begins to evaporate, resulting in a decrease in stability of both WPE and WFPE [54]. However, WFPE showed better stability compared with WPE at the same temperature. The good stability of WFPE with high temperatures suggests that it may have strong potential for applications in the field of thermally processed products.

### 3.11. Antioxidant Activities of the Emulsion

**Figure 9:**
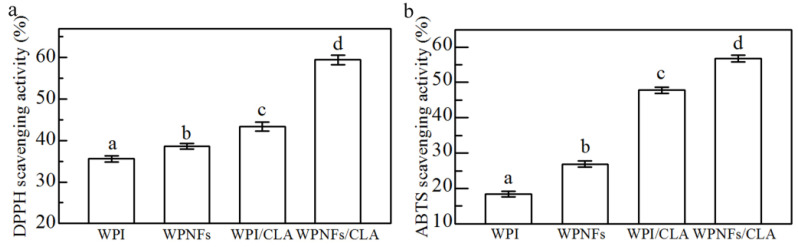
Antioxidant activity of emulsions. (**a**) 2,2-Diphenyl-1-picrylhydrazyl and (**b**) 2,2′-amino-di(2-ethyl-benzothiazoline sulphonic acid-6)ammonium salt radical scavenging activity.

Figure 9 shows the DPPH and ABTS^+^ radical scavenging activity of WPI, WPNFs, WPE and WFPE. In emulsion systems, the rate of oxidation of LBI is directly influenced by the properties of each component. The provision of electrons and neutralization of free radicals by hydroxyl groups and certain amino acid residues introduced by hydrolysis during fibrosis gives WPNFs better DPPH and ABTS^+^ radical scavenging activity than WPI [55,56,57], which was one of the reasons why WFPE had higher antioxidant activity. On the other hand, as a direct result of the inherent antioxidant activity of CLA, both WPE and WFPE showed a greater increase in antioxidant activity than before they were combined with CLA. The Pickering emulsion stabilized by WPNFs improved the solubility of CLA in water, and WFPE exhibited good ionic strength stability, thermal stability and antioxidant activity. WEPE will have broad application prospects as a food delivery system for LBI substances.

## 4. Conclusions

In this paper, WPNFs were prepared by microwave heating method using WPI as the raw material. Compared with the traditional acid method, microwave heating significantly reduced the time for fibril formation. The factors affecting the formation of WPNFs were investigated by various methods.

CLA and WPNFs were chosen to prepare stable Pickering emulsions, and the microstructure of the emulsions was evaluated using Cryo-SEM, SRM and CLSM. The results show that WPNFs were located around the oil droplets and could be used as an oil-in-water Pickering emulsifiers. Compared with WFE, the droplet appearance of WFPE is closer to a regular sphere, and the droplets are smaller. In terms of antioxidant activity, WFPE has a better ability to remove DPPH and ABTS^+^ radicals compared with WPE. In addition, WFPE is more stable in terms of droplet size and zeta potential over a wider range of ionic strengths and temperatures. Our findings suggest that WPNFs can be used as a novel carrier for CLA, improving the water solubility of CLA and providing a new solution for the defects of LBI. WFPE, as a new type of Pickering emulsion, also provides an antioxidant material for potential development in the food industry. 

## Data Availability

The data presented in this study are available on request from the corresponding author.

## Supplementary Materials

The following are available online at www.mdpi.com/article/10.3390/foods10081892/s1, Table S1: Amino acid sequence of possible building blocks of WPNFs identified by LC-MS/MS.
