# Supplementary material for: Development of Antioxidant and Stable Conjugated Linoleic Acid Pickering Emulsion with Protein Nanofibers by Microwave-Assisted Self-Assembly
Source: Foods. 2021 Aug 15;10(8):1892. doi: 10.3390/foods10081892 (PMC8391289; doi:10.3390/foods10081892)
Supplement: Supplementary file 1 [file foods-10-01892-s001.zip › foods-1311816-supplementary.pdf]

Table S1. Amino acid sequence of possible building blocks of WPNFs identified by LC-MS/MS.

Amino Acid Sequence	Molecular Weight (Da)		Source	Score
	Measured Values	Deviation		
-.LIVTQTM*KGLD.I	1234.67116	−0.00067	β-lg	23.68
-.LIVTQTM*KGLDIQK.V	1603.90875	−0.00271	β-lg	48.44
-.LIVTQTM*KGLDIQKVAGTWYSLAMAASD.I	3027.55295	0.00127	β-lg	34.04
-.LIVTQTMK.G	933.54376	0.00228	β-lg	43.8
-.LIVTQTMKG.L	990.56523	−0.00122	β-lg	42.09
-.LIVTQTMKGL.D	1103.64929	0.00231	β-lg	32.7
-.LIVTQTMKGLD.I	1218.67624	0.00491	β-lg	36.5
-.LIVTQTMKGLDIQ.K	1459.81888	0.00099	β-lg	44.85
-.LIVTQTMKGLDIQK.V	1587.91383	0.01431	β-lg	30.63
-.LIVTQTMKGLDIQKVAG.T	1815.04083	0.00177	β-lg	23.21
-.LIVTQTMKGLDIQKVAGTWYSLAMAASD.I	3027.55295	0.00394	β-lg	48.25
-.LIVTQTMKGLDIQKVAGTWYSLAMAAS.D	2896.53108	−0.00379	β-lg	29.61
-.LIVTQTMKGLDIQKVAGTWYSLAMAASD.I	3011.55803	0.00706	β-lg	54.69
-.LIVTQTMKGLDIQKVAGTWYSLAMAASDISLLD.A	3552.86918	0.00347	β-lg	22.9
A.LKALPMHIR.L	1078.65535	0.0009	β-lg	42.49
A.LNENKVLVLD.T	1156.65719	0.00227	β-lg	30.6
A.LPMHIRL.S	879.52328	0.0014	β-lg	28.75
A.MAASDISLLD.A	1035.50267	−0.0016	β-lg	22.04
A.PLRVYVEELKPTPEG.D	1726.93739	0.00934	β-lg	28.6
A.PLRVYVEELKPTPEGD.L	1841.96434	−0.00003	β-lg	25.18
A.QSAPLRVYVEELKPTPEGD.L	2128.09205	−0.00139	β-lg	28.28
C.LVRTPEVDD.E	1043.53677	−0.00147	β-lg	29.38
C.LVRTPEVDDEALEKFD.K	1875.93344	−0.00521	β-lg	26.84
D.ALNENKVLVL.D	1112.66736	0.00427	β-lg	22.7
D.ALNENKVLVLD.T	1227.6943	0.00436	β-lg	29.57
D.ALNENKVLVLDT.D	1328.74199	0.01669	β-lg	38.56
D.ALNENKVLVLDTD.Y	1443.76893	−0.00153	β-lg	36.2
D.ALNENKVLVLDTDYK.K	1734.92721	0.01131	β-lg	35.08
D.ALNENKVLVLDTDYKK.Y	1863.02217	0.00044	β-lg	39.05
D.ALNENKVLVLDTDYKKY.L	2026.08549	0.00581	β-lg	44.98
D.AQSAPLRVY.V	1004.55234	0.00315	β-lg	24.22
D.AQSAPLRVYV.E	1103.62076	0.00263	β-lg	25.48
D.AQSAPLRVYVE.E	1232.66334	0.00022	β-lg	42.37
D.AQSAPLRVYVEEL.K	1474.78999	−0.00093	β-lg	25.91
D.AQSAPLRVYVEELK.P	1602.88495	−0.00022	β-lg	39.1
D.AQSAPLRVYVEELKPT.P	1800.9854	0.00187	β-lg	27.14
D.AQSAPLRVYVEELKPTPE.G	2027.08075	−0.01138	β-lg	23.55
D.AQSAPLRVYVEELKPTPEG.D	2084.10222	−0.00163	β-lg	33.3
D.AQSAPLRVYVEELKPTPEGD.L	2199.12916	−0.00315	β-lg	31.1
D.AQSAPLRVYVEELKPTPEGDL.E	2312.21322	−0.00915	β-lg	26.74
D.DEALEKFD.K	966.44145	−0.00019	β-lg	21.27
D.DEALEKFDK.A	1094.53641	−0.00032	β-lg	21.79
D.DEALEKFDKA.L	1165.57351	0.00259	β-lg	22.04
D.DEALEKFDKALK.A	1406.75253	0.00023	β-lg	35.89
D.DEALEKFDKALKA.L	1477.78964	0.0031	β-lg	33.36
D.DEALEKFDKALKAL.P	1590.8737	−0.00276	β-lg	21.36
D.DEALEKFDKALKALPM*HIRLSFNPT.Q	2900.53383	0.04238	β-lg	28.26

D.DEALEKFDKALKALPMH.I	1956.02586	−0.00433	β-lg	43.56
D.DEALEKFDKALKALPMHIR.L	2225.21103	−0.00715	β-lg	58.27
D.DEALEKFDKALKALPMHIRL.S	2338.29509	−0.00644	β-lg	34.57
D.DEALEKFDKALKALPMHIRLS.F	2425.32712	−0.00254	β-lg	47.35
D.EALEKFDKAL.K	1163.63063	0.00314	β-lg	31.4
D.EALEKFDKALK.A	1291.72558	−0.00098	β-lg	27.63
D.EALEKFDKALKALPMH.I	1840.99891	0.00271	β-lg	27.36
D.EALEKFDKALKALPMHIR.L	2110.18408	0.00313	β-lg	31.9
D.EALEKFDKALKALPMHIRL.S	2223.26814	0.00089	β-lg	64.84
D.IQKVAGTWYSLAMAASD.I	1811.89963	0.0023	β-lg	23.69
D.IQKVAGTWYSLAMAASDISLLD.A	2353.21078	0.00268	β-lg	35.06
D.ISLLDAQ.S	759.42468	0.00248	β-lg	20.05
D.ISLLDAQSAPLR.V	1283.73175	0.00022	β-lg	80.21
D.ISLLDAQSAPLRVY.V	1545.86349	0.00337	β-lg	87.02
D.ISLLDAQSAPLRVYVE.E	1773.9745	−0.00222	β-lg	30.69
D.KALKALPMH.I	1008.60225	0.00042	β-lg	57.07
D.KALKALPMHIR.L	1277.78741	−0.00092	β-lg	53.1
D.KALKALPMHIRL.S	1390.87147	−0.00152	β-lg	44.33
D.LEILLQK.W	856.5502	0.00187	β-lg	20.03
F.KIDALNENKVLVLD.T	1583.90027	−0.00188	β-lg	25.78
K.ALKALPMHIR.L	1149.69246	0.00041	β-lg	38.41
K.ALPMHIR.L	837.47633	−0.00085	β-lg	22.83
K.IIAEKTKIPAVFKID.A	1686.01999	−0.00294	β-lg	21.66
K.IPAVFKID.A	902.53457	0.00027	β-lg	23.61
K.KIIAEKTKIPAVFKID.A	1814.11494	0.00071	β-lg	25.44
L.DAQSAPLRVYVEELKPTPEGD.L	2314.15611	−0.00178	β-lg	28.13
L.IVTQTMK.G	820.4597	0.00057	β-lg	41.23
L.IVTQTMKG.L	877.48117	0.00044	β-lg	27.12
L.KALPMHIR.L	965.57129	−0.00008	β-lg	41.58
L.RVYVEELKPTPEGD.L	1631.82751	−0.00261	β-lg	26.34
N.KVLVLDTD.Y	902.51933	0.00031	β-lg	28.18
Q.KKIIAEKTKIPAVFKID.A	1942.2099	0.0002	β-lg	20.52
Q.SAPLRVYVEELKPTPEGD.L	2000.03348	0.00043	β-lg	20.73
Q.TMKGLDIQKVAGTWYSLAMAASD.I	2457.21522	0.00086	β-lg	21.55
R.LSFNPTQLEEQCHI.-	1715.80574	−0.00611	β-lg	53.67
R.TPEVDDEALEK.F	1245.58449	0.00244	β-lg	33.89
R.VYVEELKPTPEGD.L	1475.72641	0.00168	β-lg	62.44
S.APLRVYVEELKPTPEGD.L	1913.00145	−0.0031	β-lg	40.68
D.KFLDDD.L	752.34611	0.00031	α-la	27.97
D.KFLDDDLTD.D	1081.5048	0.00034	α-la	33.02
D.KFLDDDLTDD.I	1196.53175	−0.00045	α-la	31.7
D.TQAIVQ.N	659.37227	0.00117	α-la	30.57

“-“ refers the end of the protein and there are no any other amino acids. “.” refers to the restriction site of Typsin. Mass spectrometry only detects the peptide sequence between these two points. The amino acids on both sides are next to the peptide that matches the database. Amino acid “*” refers to amino acids that may be modified.
